# Parental responses and catastrophizing in online cognitive behavioral therapy for pediatric functional abdominal pain: A mediation analysis of a randomized controlled trial

**DOI:** 10.3389/fpain.2022.962037

**Published:** 2022-10-03

**Authors:** Maria Lalouni, Aleksandra Bujacz, Marianne Bonnert, Karin B. Jensen, Anna Rosengren, Erik Hedman-Lagerlöf, Eva Serlachius, Ola Olén, Brjánn Ljótsson

**Affiliations:** ^1^ Department of Clinical Neuroscience, Karolinska Institutet, Stockholm, Sweden; ^2^ Department of Medicine, Solna, Karolinska Institutet, Stockholm, Sweden; ^3^ Stockholm Health Care Services, Region Stockholm, Stockholm, Sweden; ^4^ Department of Learning, Informatics, Management and Ethics, Karolinska Institutet, Stockholm, Sweden; ^5^ Department of Leadership and Command / Control, The Swedish Defense University, Stockholm, Sweden; ^6^ Department of Medical Epidemiology and Biostatistics, Karolinska Institutet, Stockholm, Sweden; ^7^ Department of Clinical Neuroscience, Centre for Psychiatry Research, Karolinska Institutet / Stockholm Health Care Services, Region Stockholm, Stockholm, Sweden; ^8^ Department of Clinical Sciences, Faculty of Medicine, Lund University, Lund, Sweden; ^9^ Department of Paediatric Gastroenterology and Nutrition, Sachs’ Children’s Hospital, Stockholm, Sweden

**Keywords:** functional abdominal pain, parents, children, irritable bowel syndrome, mediation analysis

## Abstract

**Objective:**

To test if decreased parental protective behaviors, monitoring behaviors, and parental catastrophizing mediate relief of gastrointestinal symptoms in children 8–12 years with functional abdominal pain disorders (FAPDs). The study uses secondary data analyses of a randomized controlled trial in which exposure-based online cognitive behavioral therapy (ICBT) was found superior to treatment as usual in decreasing gastrointestinal symptoms.

**Methods:**

The ICBT included 10 weekly modules for children and 10 weekly modules for parents. Treatment as usual consisted of any medication, dietary adjustments, and healthcare visits that the participants engaged in during 10 weeks. All measures were self-assessed online by parents. Biweekly assessments of the Adult Responses to Children's Symptoms (ARCS), Protect and Monitor subscales, and the Pain Catastrophizing Scale, parental version (PCS-P) were included in univariate and multivariate growth models to test their mediating effect on the child's gastrointestinal symptoms assessed with the Pediatric Quality of Life Gastrointestinal Symptoms Scale (PedsQL).

**Results:**

A total of 90 dyads of children with FAPDs and their parents were included in the study, of which 46 were randomized to ICBT and 44 to treatment as usual. The PCS-P was found to mediate change in the PedsQL *ab* = 0.639 (95% CI 0.020–2.331), while the ARCS Monitor *ab* = 0.472 (95% CI −1.002 to 2.547), and Protect *ab* = −0.151 (95% CI −1.455 to 0.674) were not mediators of change.

**Conclusions:**

To target parental catastrophizing in ICBT for pediatric FAPDs is potentially important to reduce abdominal symptoms in children.

## Introduction

Pediatric functional abdominal pain disorders (FAPDs) are highly prevalent (1) and characterized by recurrent or persistent abdominal pain (2). FAPDs are associated with low health-related quality of life (3) and psychiatric comorbidity (4–6), and for many of the affected children, the symptoms continue into adulthood (7). FAPDs have been shown to be aggregated within families, which can be explained by genetic predisposition, but also by social learning (e.g., children may learn how to respond to abdominal symptoms through observation of their parents’ behavior) (6, 8).

When children are in pain, most parents will do almost anything to ease their suffering. It may be intuitive for parents to use protective and monitoring behaviors to help their children to avoid challenging situations and painful symptoms, even when the symptoms are not harmful, as in FAPDs. Protective behaviors include allowing the child to stay home from school or to do the child's chores. Monitoring behaviors include a check on the child or to ask how the child feels. To use protective and monitoring behaviors is a natural form of parenting, but when used extensively, they may increase the child's avoidance of symptoms and challenging situations (e.g., school). Such avoidance often results in short-term relief of symptoms, but in the long run, it feeds into a vicious circle of fear and avoidance, maintaining symptoms and disability (9, 10). Therefore, decreasing fear and avoidance is a key treatment target in psychological treatments for FAPDs (11–13), and in pediatric FAPDs, it is also important to address parental behaviors (4, 14, 15). Parents may not only remind their child about assignments, facilitate exercises, and reinforce efforts, but also help decrease avoidance of symptoms and encourage engagement in important areas of life (e.g., school, leisure activities, being with friends) (4, 5, 16, 17).

In a literature review, Newton et al. reported that parents’ catastrophic thoughts about their child's pain were associated with an increased tendency for the child to report both abdominal and other bodily symptoms (18). Such catastrophic thoughts may include worrying about whether the child's pain will ever end or thinking about one's inability to make the child's pain go away. Parents who engage in catastrophic thoughts may be more prone to notice and react to their child's symptoms compared with parents who do not catastrophize. Such reactions (e.g., protective and monitoring behaviors) may increase the child's perception of pain. In a classic experiment, Walker and coauthors examined different parental responses to experimentally induced abdominal pain. They found that parental attention to pain increased children's pain complaints and also their pain ratings after the experiment (19). Thus, parental protective and monitoring behaviors, and catastrophic thoughts, may increase pain and other gastrointestinal (GI) symptoms in the child.

**Figure 1:**
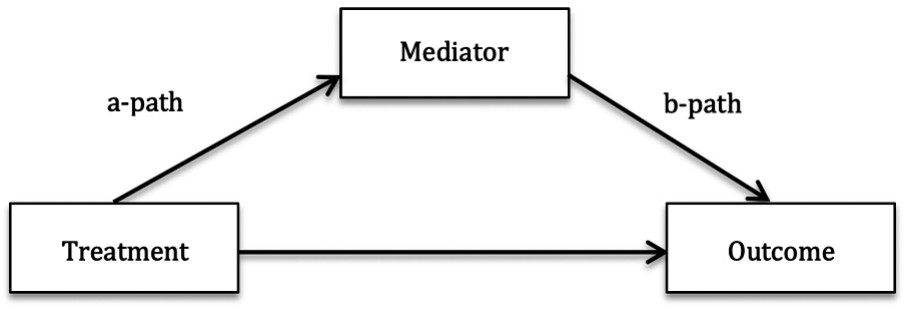
Mediation model illustrating the direct effect of the treatment on the outcome, and the mediated, or indirect, effect on the outcome, the *ab*-path.

Mediation analysis can be used to study trajectories of process and outcome measures during treatment and may reveal mechanisms involved in symptom relief. A mediation model assesses if some of a treatment's effect on an outcome variable is mediated *via* a change in another variable, i.e., a mediator (Figure 1). We have previously shown that a reduction in children's GI-specific avoidance behavior (11, 12) and GI-specific anxiety (12), but not stress (11), mediates gastrointestinal symptom relief in exposure-based online cognitive behavioral therapy (ICBT) for children and adolescents with FAPDs. Despite the fact that parental behavior is considered to be an important treatment target to minimize children’s pain, only two prior mediation studies have assessed parental process variables in relation to children's symptom relief or function (20, 21). Levy et al. investigated mechanisms of change in a brief cognitive behavioral intervention for children with FAPDs and their parents, using data from pre-treatment, post-treatment, and follow-up. They found that reductions in parents’ perceived threat regarding their child's pain mediated the child's pain intensity at follow-up (20). Van Tilburg et al. also tested a brief cognitive behavioral intervention and found that decreased parental catastrophizing mediated improvements in children's quality of life, healthcare utilization, missed school, and disability, and that decreased parental protectiveness mediated child disability and missed school (21). Process and outcome variables were not assessed repeatedly during treatment in the studies by Levy and Van Tilburg, and Levy et al. recommend expanded measurement points to provide more detail on the mediational processes (20).

The current study uses repeated measures by parents assessed during a randomized controlled trial (RCT) in which exposure-based ICBT was found superior to treatment as usual in children with FAPDs (5). The objective was to assess whether a reduction of parental protective and monitoring behaviors and parental catastrophic thoughts mediated improvement in child's gastrointestinal symptoms in ICBT compared with treatment as usual.

## Methods

The data in this study were collected in an RCT (Clinicaltrials.gov, August 2016, NCT02873078) approved by the Regional Ethical Board in Stockholm, Sweden August 2016 (2016/1289-31) (5). The patients were consecutively referred to the study between September 2016 and April 2017, and the study site was the Child and Adolescent Psychiatry Research Center in Stockholm. The center is an outpatient research clinic within the Child and Adolescent Mental Health Services in Stockholm, Sweden, and has a specific focus on research and development of psychological treatments *via* the Internet. In Sweden, many pediatric gastrointestinal units do not have access to their own psychologists. After the study was completed, a regular Internet unit within the Child and Adolescent Mental Health Services in Stockholm was opened and children with FAPDs are now being referred there instead.

### Participants

Ninety children with FAPDs and one of their parents were included in the study. The inclusion criteria for the children were as follows: (a) ≥8 and ≤12 years old; (b) diagnosed according to the ROME IV criteria with at least one of the following FAPDs: irritable bowel syndrome, functional dyspepsia, or functional abdominal pain, not otherwise specified (2); (c) if using psychopharmacological medications, the dose had to be stable for at least a month; (d) access to the Internet; (e) writing and reading Swedish (child and parent). The exclusion criteria were as follows: (f) another somatic disorder that could explain their symptoms; (g) social or psychiatric disorder that could explain their symptoms; (h) school absenteeism >40% (deemed to need a more comprehensive support instead of online treatment); or (i) ongoing psychological treatment.

### Procedure

All children were referred to the study by physicians who certified the FAPD diagnosis. Before inclusion, children and parents met a psychologist who assessed the child's psychiatric comorbidity with the MINI-KID psychiatric interview (22) and the inclusion/exclusion criteria. The self-rated questionnaires were assessed online in the families’ homes before, during, and after ICBT or treatment as usual, without the influence of study personnel. Participants were enrolled by ML who provided anonymous study IDs to the randomizer, an independent researcher not involved in the study. Randomization to ICBT (*n* = 46) or treatment as usual (*n* = 44) was conducted in cohorts (sizes 5–19, balanced 1:1 within blocks). The sizes of the cohorts varied depending on how many children had been recruited at prespecified time points. A list randomizer at www.random.org was used to generate the allocation sequence for each cohort. The participants were not blinded as to which groups they were assigned to. For a thorough description of the study procedure, see the main article (5).

### Interventions

#### ICBT

ICBT is an online exposure-based cognitive behavioral therapy in which children and parents engaged in 10 weekly modules each. Parents also took part in their child's modules together with the child. Communication with the therapist was asynchronous and text-based within the treatment platform.

The child modules are based on exposure for abdominal symptoms and situations in which symptoms are perceived as particularly difficult. Exposure exercises were chosen by the families with help from the therapist and conducted between the modules. Examples of exposure exercises were eating something that cause abdominal symptoms, being in school or engaging in leisure activities with symptoms, postponing toilet visits, or leaving “just in case” medication at home when going out.

In the parental modules (Table 1), parents learned that although overly protective behaviors are understandable, they are not helpful in the long run. Instead, the parents were taught to encourage and facilitate the child's exposures and to redirect attention from abdominal pain to other important areas in the child's life (school, friends, play, etc.). The parents learned to first validate their child's experience and then to help their child to shift focus. To increase parental focus on activities and decrease focus on the stomach, “golden moments” were scheduled. During these moments, the parent and child engaged in an activity chosen by the child. The parent gave full attention to the child, without focusing on the stomach. For a thorough description of the treatment, see Lalouni et al. (4, 23).

**Table 1:** Overview of the parental modules.

Module 1	The role of parental attention in relation to the child's focus on symptoms. Validating the child's symptoms and shifting focus.
Module 2	Golden moments as a means to increase attention to healthy behaviors and decrease attention to the stomach.
Module 3	Encouragement of the child's exposure exercises including a game chart. Cooperating with school.
Module 4	Handling stressful events. Planning own recreational activities.
Module 5	Review of modules 1–4.
Module 6	Problem solving together with the child.
Module 7	Functional analysis of the own behavior with focus on the child–parent interaction.
Module 8	Review of the treatment, part 1. Rewarding yourself for work well done.
Module 9	Review of the treatment, part 2. Lessons learned and remaining parental challenges.
Module 10	Review of parental behaviors. Maintenance and relapse prevention.

#### Treatment as usual

Treatment as usual included any other treatment that the families engaged in during the 10-week intervention period. It included healthcare visits, medication, and dietary adjustments. Such treatments were also allowed in the ICBT group. The children in the treatment as usual group used significantly more healthcare resources compared with the children in the ICBT group. A detailed description of the interventions in both groups is provided in the original report of the RCT (5).

### Measures

#### Outcome variable

The Pediatric Quality of Life Gastrointestinal Symptom Scale (PedsQL) was used as the outcome variable. It was developed for children with FAPDs, and in the present study, the parent report of the scale was used. The PedsQL contains nine items that assess different abdominal symptoms on a 5-point scale. The PedsQL is transformed to a 0–100 scale, in which low values indicate high symptom severity and high values indicate mild symptoms. The PedsQL has shown acceptable internal consistency (Cronbach's *α* 0.77) (24) and is sensitive to change (4, 5, 23). In this sample, Cronbach's *α* was 0.72 at baseline.

#### Proposed mediators

The proposed mediators were *parental responses to their child's symptoms*, assessed with the Adult Responses to Children's Symptoms (ARCS), Protect and Monitor subscales (25, 26), and *parental catastrophizing about their child's symptoms*, assessed with the Pain Catastrophizing Scale, parental version (PCS-P) (27). The ARCS Protect and Monitor subscales have been shown to be responsive and sensitive to change (26, 28). In this sample, Cronbach's *α* was 0.82 for subscale Protect and *α* 0.81 for subscale Monitor at baseline. The PCS-P has been shown to significantly contribute to the explanation of children's disability and school attendance (27) and is sensitive to change (5). In this sample, Cronbach's *α* was 0.90 for the PCS-P at baseline.

#### Assessments

The PedsQL was assessed weekly during the treatment (weeks 1–10). At baseline (week 0) and post assessments (week 11), a version of the PedsQL with a 1-month recall period (instead of a 1-week one) was used. Therefore, only the weekly assessments were used in the analysis. The ARCS and PCS-P were assessed biweekly in a wave missing design (planned missing). This design was chosen so that parents would not be overloaded by questions and thereby provide less reliable assessments. To be able to assess all questionnaires all weeks, half of the parents in each condition were randomized to assess the ARCS on weeks 1, 3, 5, 7, and 9 and the PCS-P on weeks 2, 4, 6, 8, and 10. The other half in each condition assessed the ARCS on weeks 2, 4, 6, 8, and 10 and the PCS-P on weeks 1, 3, 5, 7, and 9. Both groups assessed the ARCS and PCS-P on weeks 0 and 11.

### Statistical analysis

The power calculation revealed that at least 80 participants were needed to achieve 80% power to detect a between group effect size of Cohen's *d* = 0.6 (*α* 0.05) on the main outcome in the randomized controlled trial. We considered the present mediation analyses to be exploratory and thus did not perform *a priori* power calculations for them. Univariate and multivariate growth models with random effects were used to test mediation hypotheses with three proposed mediators: Adult Responses to Children's Symptoms subscales Monitor and Protect, as well as parent assessed pain catastrophizing (PCS-P). All available data were used to model change over time. Because there was a deaccelerating rate of change over the treatment period, growth rates were modeled as a function of the square roots of weeks (29). The analyses were conducted in Mplus version 8.4 (30). First, univariate growth models evaluating the effect of treatment were estimated for the outcome variable, as well as the three mediators. Second, these univariate models were combined into parallel process growth models, including the outcome and one mediator per each model. If mediators were shown to be statistically significant, a reversed analysis, using the outcome measure (PedsQL) as mediator, and the mediator as outcome variable, was conducted to investigate possible reverse causation effects. Inferences about statistical significance of the estimates in the parallel process mediation models, i.e., *a*- and *b*-paths and their end product, *ab*, which is the indirect or mediated effect, were based on bootstrapped 95% confidence interval (5,000 replications), meaning that confidence intervals that did not include zero were considered statistically significant. Blinding was not used in the statistical analysis.

## Results

A total of 90 dyads of children with FAPDs and one of their parents were included in the study, of which 46 were randomized to ICBT and 44 to treatment as usual. Most children were girls 62/90 (69%), and their average age was 10.2 (SD = 1.4). Most parents were mothers 77/90 (86%). Baseline and clinical characteristics are presented in Table 2. Children completed a mean of 9.3 of their 10 weekly modules and parents completed a mean of 9.2 of their 10 weekly parental modules. No child or parent completed fewer than five modules. All available data were included in the analysis. No serious adverse events were reported during the study. Transient undesirable effects in both groups are reported in detail in the original study (5). The proportion missing data in the sample was 4%, not including the planned missing data of the proposed mediators, which was 50% according to the design. Descriptive statistics for the outcome variable and the proposed mediators are presented in Table 3.

**Table 2:** Demographic and clinical characteristics (*n* = 90).

	Internet-CBT (*n* = 46)	Treatment as usual (*n* = 44)
Children's characteristics		
Age, mean (SD)	10.1 (1.2)	10.4 (1.5)
Gender, *n* (%) female	28 (61%)	34 (77%)
Duration of abdominal symptoms, years, mean (SD)	3.4 (2.0)	3.9 (2.2)
Parents’ characteristics		
Gender parents, *n* (%) female	39 (85%)	38 (86%)
Baseline clinical assessment		
PedsQL, mean (SE)	59.90 (2.06)	55.74 (2.11)
ARCS Monitor, mean (SE)	10.83 (0.50)	12.23 (0.51)
ARCS Protect, mean (SE)	10.11 (0.93)	11.55 (0.95)
PCS-P, mean (SE)	16.28 (1.38)	21.02 (1.41)

CBT, cognitive behavioral therapy; PedsQL, Gastrointestinal Symptom Scale measuring abdominal symptoms; ARCS Monitor, Adult Responses to Children's Symptoms, subscale monitor; ARCS Protect, Adult Responses to Children's Symptoms, subscale protect; PCS-P, Pain Catastrophizing Scale, parent version; SD, standard deviation; SE, standard error.

**Table 3:** Number of observations, observed means, standard deviations, and missing observations for weekly measures of outcome measure and proposed mediators.

Time point	PedsQL			ARCS Monitor			ARCS Protect			PCS-P		
	*n*	*m*	SD	*n*	*m*	SD	*n*	*m*	SD	*n*	*m*	SD
0	—	—	—	90	11.51	3.42	90	10.81	6.91	90	18.60	9.30
1	89	66.39	14.67	45	10.98	2.99	45	10.02	7.32	45	18.96	10.87
2	86	69.83	12.94	45	8.82	3.73	45	8.53	5.99	44	16.98	9.53
3	85	69.35	15.43	45	8.44	4.19	45	8.87	8.29	45	13.67	10.28
4	85	71.99	14.94	45	7.00	4.20	45	7.09	6.13	45	14.98	10.03
5	88	71.24	16.27	45	7.96	4.38	45	7.11	8.19	45	12.13	10.55
6	81	72.12	13.02	45	6.27	4.25	45	5.87	5.67	44	14.00	9.75
7	84	72.85	14.08	45	7.53	4.75	45	7.40	9.18	44	10.98	10.10
8	78	72.86	15.47	42	5.97	4.47	42	5.74	6.83	45	12.42	9.85
9	82	73.44	15.11	44	7.25	4.97	44	5.68	8.28	41	10.90	11.30
10	83	74.97	16.14	37	5.46	4.38	37	4.84	6.33	37	12.57	11.13
11	—	—	—	87	6.01	4.23	87	4.67	5.82	87	11.05	10.73
Total obs	841			615			615			612		
Missing obs	59			15			15			18		

PedsQL, Gastrointestinal Symptom Scale measuring abdominal symptoms; ARCS Monitor, Adult Responses to Children's Symptoms, subscale monitor. ARCS Protect, Adult Responses to Children's Symptoms, subscale protect; PCS-P, Pain Catastrophizing Scale, parent version; n, number of observations; m, mean; SD, standard deviation.

The univariate growth model showed that the average trajectories of the outcome measure (PedsQL) differed between treatment conditions: estimate = 1.863, SE = 0.811, *p* = 0.022 (standardized estimate = 0.721, SE = 0.339, *p* = 0.034). The difference between treatment conditions for mediators (i.e., *a-*paths) calculated in the univariate growth models was also significant and is as follows: ARCS Monitor estimate = −1.122, SE = 0.237, *p* < 0.001 (standardized estimate = −1.026, SE = 0.183, *p* < 0.001); ARCS Protect estimate = −0.906, SE = 0.358, *p* = 0.011 (standardized estimate = −0.601, SE = 0.225, *p* = 0.008); and PCS-P estimate = −1.242, SE = 0.605, *p* = 0.040 (standardized estimate = −0.475, SE = 0.223, *p* = 0.033).

The main results of testing mediation hypotheses in the parallel process growth models are presented in Table 4. Consistent with the univariate models, all *a*-paths in the joint models (i.e., the effects of treatment on the growth slope of a mediator) were statistically significant. None of the *b*-paths (i.e., the regression of the outcome growth slope on the mediator growth slope) were significant, and the *ab*-product (i.e., mediated effect) of the ARCS subscales was also not significant when evaluated with 95% bootstrapped confidence intervals. However, the *ab*-product for the PCS-P scale was significant, *ab* = 0.639 (95% CI 0.020–2.331). When a reverse mediation path was evaluated, with the PCS-P growth slope treated as an outcome and PedsQL growth slope acting as a mediator, the *ab*-path was not statistically significant (*ab* = 0.376, 95% CI −0.481 to 3.607).

**Table 4:** Unstandardized estimates from the parallel process mediation models.

Mediator	*a*-path		*b*-path		*ab*-path	
	Estimate	95% CI	Estimate	95% CI	Estimate	95% CI
ARCS Monitor	−1.238^a^	(−1.694 to −0.754)	−0.381	(−1.805 to 0.906)	0.472	(−1.002; 2.547)
ARCS Protect	−0.897^a^	(−1.566 to −0.185)	0.168	(−0.794 to 1.406)	−0.151	(−1.455 to 0.674)
PCS-P	−1.651^a^	(−2.916 to −0.282)	−0.387	(−0.853 to 0.025)	0.639*	(0.020 to 2.331)

95% CI, 95% confidence interval based on 5,000 bootstrap replications; ARCS Monitor, Adult Responses to Children's Symptoms, subscale monitor; ARCS Protect, Adult Responses to Children's Symptoms, subscale protect; PCS-P, Pain Catastrophizing Scale, parent version.

^a^ Denotes statistical significance by a 95% CI that does not include zero.

## Discussion

In this study, we tested whether reductions of parental protective behaviors, parental monitoring behaviors, or parental catastrophizing mediated improvements in gastrointestinal symptoms for children with FAPDs in an exposure-based ICBT compared with treatment as usual. The results showed that parental catastrophizing, but not parental protective or monitoring behaviors, was a mediator of change for children in the ICBT. Further, the reversed analysis, in which reduced gastrointestinal symptoms in children was tested as a mediator for reduced parental catastrophizing, was not significant. This is an important distinction, because it can be hypothesized that when a child's symptom decreases, it could lead to parents feeling less anxious and thus less prone to catastrophize about the child's symptoms. However, our results rather indicate that when parents manage to catastrophize less, their children's symptoms decrease. These results corroborate the findings by Levy and coauthors, in which reductions in parents’ perceived threat regarding their child's pain mediated a reduction in gastrointestinal symptom severity at follow-up (20), and the results by van Tilburg et al., showing that reduced parental catastrophizing mediated functional outcomes (e.g., child disability and healthcare utilization) at follow-up (21). Also, Caes et al. showed in an experimental pain study including both children with chronic pain and healthy children that parents in both groups with high catastrophic thinking experienced a greater tendency to stop their child's pain experiment (31).

In an exposure-based ICBT, the parents’ catastrophic thoughts are not directly challenged with cognitive interventions. Instead, parental behaviors, such as redirecting the attention from the child's abdominal symptoms to other important areas of the child's life, are taught to the parents. These behaviors are more in line with decreased monitoring and protective behaviors. However, they may also work as exposure exercises for parents’ worry about their child's symptoms. Parents oftentimes do not believe that their child will be able to cope with the symptoms. When parents gain new experiences that another approach is possible and even helpful for their child, their cognitions about their child's ability to cope are challenged. So, even though the intervention did not use cognitive techniques to change catastrophizing, we suggest that it is justified to use a mediation model rather than a moderation model as it can be assumed that (a) change in parental behaviors occurred because of the treatment, (b) parental behaviors affect catastrophizing, and (c) reduced catastrophizing leads to symptom improvement. Analogously, even if we do not explicitly work with the child's cognitions, these may also change when the child gains experience of being able to cope. In fact, exposure is the treatment of choice for clinical anxiety (32).

FAPDs tend to run in families (33), which can be explained by both a shared genetic predisposition for developing FAPDs and by social influence. A study of monozygotic and dizygotic twins showed that heredity contributes to the development of FAPDs (8). Further, by comparing the occurrence of FAPDs in twins (6.7%) and mothers (15.2%) of dizygotic twins with FAPDs, the authors also showed that the occurrence of FAPDs is influenced by social learning *via* parents (mothers in this study) (8). The prevailing explanatory model for FAPDs is the biopsychosocial model (6), in which symptoms are explained by an interplay of biological, psychological, and social factors. For children of younger ages, parents are the most important social role models. Parental behaviors and attitudes have been shown to constitute a major influence on their children's wellbeing (34, 35), and the fact that they spend a lot of time together allows for repeated and ongoing learning. In our previous studies, we have shown that it is possible to address and change parental protective and monitoring behaviors (4, 5, 17, 23) and catastrophizing (5) using our treatment protocol in children and adolescents with FAPDs. This study adds a mechanistic perspective to this knowledge, namely that, of the three proposed mediators, it is a reduction in parental catastrophizing that is linked to improvements in children's symptoms. Thus, parental catastrophic thoughts are likely an important treatment target in pediatric FAPDs.

Another benefit of addressing parents in treatments for children's symptoms is that, after therapy, parents will continue to influence their children for many years. To decrease parental catastrophizing will therefore likely have an effect of the child's wellbeing well beyond the time of the treatment. The results of this study can be relevant also in other pediatric conditions in which parental catastrophizing may influence children's symptoms and fear of symptoms. In fact, Langer et al. (36) showed that parental catastrophizing mediated the association between child pain behaviors and parental protective behaviors in patients with inflammatory bowel disorder.

It can be argued that parents’ more extensive experience determining whether a stimulus is dangerous or innocuous is vital for a child's survival. As such, parental catastrophizing may act as an important signal that activates the child's fear and avoidance, which in longstanding pain conditions have been shown to maintain symptoms and disability (9, 10). Catastrophizing could be hypothesized as being behaviorally expressed in the other two proposed mediators, ARCS subscales Protect and Monitor. However, these behaviors may be expressed without the “valence of anxiety,” manifested in parental catastrophizing. “Lack of anxiety” may thus be one explanation as to why the changes in parental monitoring and protective behavior did not mediate improvement in the child's gastrointestinal symptoms. We have previously shown that decreased gastrointestinal-specific fear and avoidance in children mediates the relief in gastrointestinal symptoms in ICBT compared with treatment as usual (12). Future studies should explore the interplay between parental catastrophizing, symptom-specific fear and avoidance in children, and children's gastrointestinal symptoms.

The strengths of the study include the randomized study design and the use of repeated measures during treatments. A limitation was the rather small sample size, which is why the findings should be interpreted with caution. The planned missing design, in which only half the parents rated each proposed mediator every week, was both a strength and a limitation. It reduced power compared with if all parents rated all assessments every week, but on the other hand, it decreased the parents’ workload and may thereby increase the reliability of the assessments. Another limitation is that only parents’ assessments were used in the study. Children did assess their own symptoms and these results were in line with those of the parents, but with a smaller effect size (5). Because of the limited sample size, the parents’ assessments of the primary outcome measure were therefore used to optimize the power of the analyses. It could also be argued that by using the same informant for all measures, additional sources of measurement error were reduced.

In this study, we showed that reduced parental catastrophizing mediated a reduction of gastrointestinal symptoms for children in ICBT. We conclude that parental catastrophizing is potentially an important treatment target in ICBT for pediatric FAPDs.

## Data Availability

The datasets presented in this article are not readily available because we do not have ethical permit to share the data. Requests to access the datasets should be directed to maria.lalouni@ki.se.
